# Increase of nitrous oxide-induced neurological disorders – a German multicenter experience

**DOI:** 10.1186/s42466-024-00361-0

**Published:** 2025-01-16

**Authors:** Julius Nicolai Meißner, Janina Neuneier, Iason Bartzokis, Mathias Rehm, Ahmad Al-Hayali, Marcus Müller, Sebastian Paus, Volker Limmroth, Gereon R. Fink, Gabor C. Petzold, Louisa Nitsch

**Affiliations:** 1 Department of Vascular Neurology, University Hospital Bonn, Bonn, Germany; 2 Department of Neurology, University Hospital Cologne, Cologne, Germany; 3 Department of Neurology, Hospital Cologne Merheim, Merheim, Germany; 4 Department of Neurology, GFO Hospital St. Johannes Sieglar, Sieglar, Germany; 5 Department of Neurology, St. Marien-Hospital Hamm, Hamm, Germany; 6 Cognitive Neuroscience, Institute of Neuroscience and Medicine (INM-3), Research Center Jülich, Jülich, Germany; 7 Department of Neuroimmunology, University Hospital Bonn, Bonn, Germany; 8 Department of Vascular Neurology, Venusberg Campus 1, 53127 Bonn, Germany

**Keywords:** Laughing gas, N_2_O, Neurological complications, Neuropathy, Myelopathy, Vitamin-B_12_, Subacute combined degeneration

## Abstract

**Background:**

Nitrous oxide (N₂O), commonly known as laughing gas, is widely recognized for its anesthetic and analgesic effects, and is frequently used in medical contexts. However, its misuse can lead to significant neurological complications, which are often under-recognized in clinical practice. Recent data on such cases in Germany are rare. We here report the spectrum of neurological complications associated with the recreational use of N₂O, as encountered in German neurology centers.

**Methods:**

We retrospectively analyzed of 23 cases presenting with neurological symptoms following N₂O abuse between July 2020 and August 2024 across five neurology departments in Germany. Data were collected on patient demographics, clinical manifestations, diagnostic findings, and treatment approaches.

**Results:**

Over the last four years the number of cases increased. Clinical presentations primarily included neuropathy, found in all patients, along with myelopathy. The most common symptoms were sensory loss, ataxia, and motor deficits.

**Conclusion:**

Our data suggest that N₂O abuse is on the rise in Germany. Further initiatives are warranted to raise awareness among users, healthcare and professionals.

## Background

Nitrous oxide (N₂O), commonly known as laughing gas, is a colorless, non-flammable gas with significant medical and recreational uses. It is used for its anesthetic and analgesic effects during surgical procedures, childbirth and in dentistry [22]. Besides, it is consumed recreationally for its euphoric effects. Despite its widespread use [8], the potential neurological complications associated with both acute and chronic exposure to N₂O remain underappreciated, thereby increasing the risk of misdiagnosis and delayed treatment. Recent studies have underscored the neurological risks associated with N₂O, mainly when used outside controlled medical settings. Chronic exposure to N_2_O has been linked to a variety of neurological symptoms ranging from mild sensory disturbances to severe, irreversible neurological damage [15]. The underlying pathophysiology of these effects primarily involves the inactivation of vitamin-B_12_, an essential cofactor in nerve function and blood formation. This inactivation interferes with methionine synthase activity, leading to a cascade of biochemical failures that prominently affect the nervous system [4, 21].

The increase in the recreational use of N₂O in European countries has been accompanied by a parallel rise in cases of neurological complications, including subacute combined degeneration (SCD) of the spinal cord and peripheral neuropathies. The symptoms can range from numbness and tingling to more severe cases involving paralysis and cognitive changes [1]; Mair, Paris, et al., [10]). The available recent data from Germany is limited to single case reports [2, 12]. This article provides a comprehensive overview of the neurological sequelae associated with N₂O exposure observed in German neurology departments. The study´s main objective was to describe and analyze the neurological symptoms observed in 23 patients treated in five German hospitals between July 2020 and August 2024 following N₂O inhalation.

## Methods

This retrospective study included data from all consecutive patients with known N₂O inhalation and neurological disorders treated in five different German neurology departments between July 2020 and August 2024. All data were derived from existing medical records. The data set included patient characteristics, neurological symptoms, imaging results, laboratory data, and, if available, treatment and outcome. *Python* was used for calculation of mean, standard deviation and odds ratio. Figure two was created with *BioRender*.

## Results

**Fig. 1:**
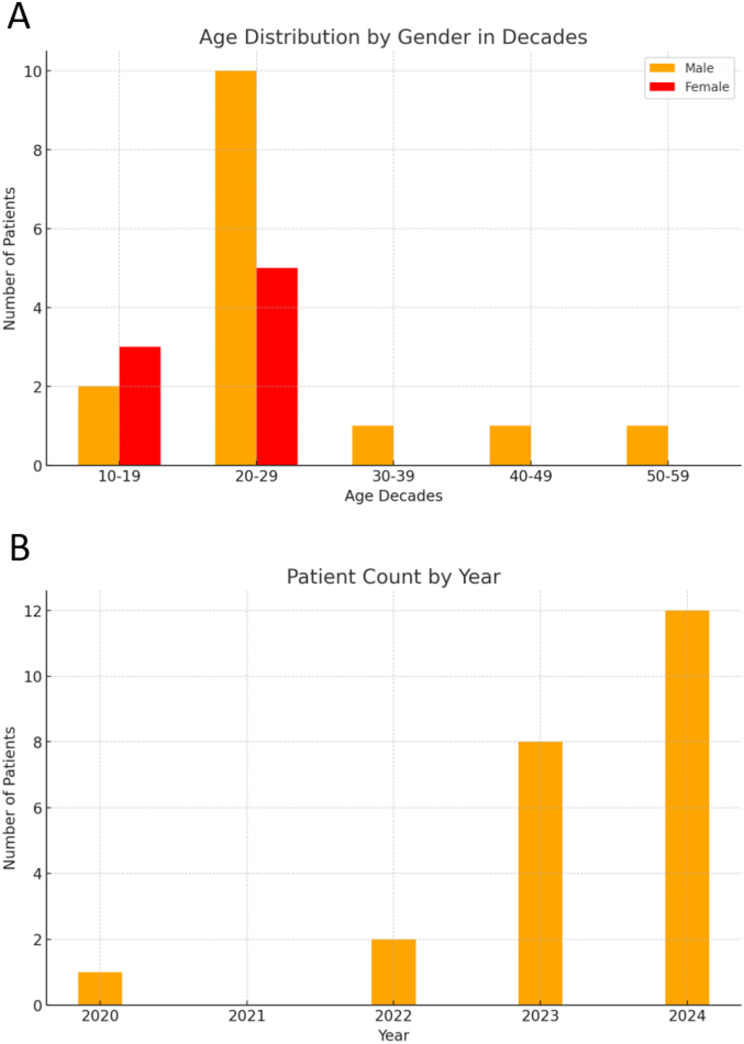
Patient characteristics. **A**: Age and Sex Distribution. **B**: Patient Count by Year

A total of 23 patients were treated for neurological symptoms induced by N₂O between July 2020 and August 2024 at the five German hospitals. All patients reported the recreational use of N₂O. Approximately two-thirds of the patients were male, with a mean age of 24.9 years (Fig. 1A). Notably, a pronounced increase in the number of cases was evident in recent years in these centers (Fig. 1B). Risk factors for malnutrition were reported in four patients (heavy drinking in two patients, celiac disease in one patient and gastric surgery in one patient).

The most frequently observed sensory symptoms were paresthesia/hypesthesia (95.7%) and abnormal pallesthesia (88.5%) (Table 1). Gait disturbances and ataxia were detected in 82.6% of patients. Additionally, limb weakness and hyporeflexia, were encountered in over 50% of patients. Autonomic symptoms were observed in only 13% of cases. Autonomic symptoms were erectile dysfunction in one patient and bladder dysfunction in one patient. One patient had autonomic symptoms that were additionally attributed to alcohol withdrawal. Other, less frequent symptoms were signs of spasticity and a positive Lhermitte’s (Table 2). One patient experienced a transient loss of consciousness (TLOC) before admission. The exact dosage of N₂O inhaled was rarely reported, with values ranging extensive daily exposures to single uses. Fifteen out of eighteen patients with available data reported regular consumption. The frequency of consumption in patients with regular abuse was only reported in a few patients (seven with daily exposure, three with weekly or twice per week exposure).

**Table 1:** Relevant results of clinical examination, apparative diagnostics and laboratory results

	Mean (SD)	Pathologic findings, *n* (%)
**Clinical examination**		
Paresthesia/Hypesthesia		22/23 (95.7%)
Ataxia		19/23 (82.6%)
Paresis		12/23 (52.2%)
Hyporeflexia		17/23 (73.9%)
Abnormal pallesthesia		17/19 (88.5%)
Autonomic dysfunction		3/23 (13.0%)
Other symptoms		7/23 (30.0%)
**Apparative Diagnostics**		
MRI abnormalities		15/21 (71.4%)
Myelopathy		14/21 (66.7%)
Neuropathy		20/20 (100%)
SSEP abnormal		10/12 (83.3%)
**Laboratory Results (reference range)**		
Vitamin-B_12_ (200–1000 pg/ml)	349.74 (209.53)	8/23 (34.8%)
Homocysteine (3.2–10.7 µmol/l)	73.01 (43.51)	8/9 (88.9%)
Methylmalonic acid (< 32 ng/ml)	475.38 (511.47)	18/19 (94.7%)
Holotranscobalamin (35–150 pmol/l)	101.42 (36.88)	5/15 (33.3%)
Folate (4.5–32.2 ng/ml)	9.23 (4.98)	3/19 (21.1%)
Hb (> 12 g/dl in females and > 13 g/dl in males)	13.68 (1.64)	6/23 (26.1%)
MCV (80-100 fl.)	92.68 (5.88)	4/23 (17.4%)
MCHC (31.5–36 g/dl)	34.09 (1.00)	0/23 (0%)
CSF protein (150–650 g/l)	0.41 (0.17)	1/18 (5.6%)
CSF cell count (≤ 5/µl)	2.47 (1.76)	1/19 (5.3%)
CSF lactate (1.1–2.4 mmol/l)	1.94 (0.40)	2/17 (11.8%)

**Table 2:** Clinical examination findings on individual patient level

Patient	Paresthesia/ Hypesthesia	Ataxia	Paresis	Hyporeflexia	Abnormal pallesthesia	Autonomic dysfunction	Other symptoms
1	yes	yes	no	no	yes	no	Lhermitte
2	yes	yes	no	yes	yes	no	no
3	yes	yes	no	yes	NA	no	no
4	yes	yes	yes	yes	yes	no	no
5	yes	yes	yes	yes	NA	no	no
6	yes	yes	no	no	yes	no	Lhermitte
7	yes	yes	no	yes	yes	no	no
8	no	yes	yes	yes	no	yes	spasticity
9	yes	yes	no	no	yes	no	no
10	yes	yes	yes	no	yes	yes	no
11	yes	no	no	no	yes	no	no
12	yes	yes	yes	no	yes	no	spasticity
13	yes	no	yes	yes	NA	no	no
14	yes	yes	yes	yes	NA	no	no
15	yes	yes	yes	yes	yes	no	no
16	yes	yes	yes	yes	yes	no	no
17	yes	yes	yes	yes	yes	no	no
18	yes	no	no	no	yes	no	no
19	yes	yes	yes	yes	yes	no	no
20	yes	yes	yes	yes	yes	no	no
21	yes	no	no	no	no	no	TLOC
22	yes	yes	no	yes	yes	no	spasticity
23	yes	yes	no	yes	yes	yes	Lhermitte

Magnetic resonance imaging was conducted in most patients (Table 1), revealing evidence of myelopathy in approximately two thirds of cases. The precise spinal cord level affected was identified by magnetic resonance imaging in nine patients. In all of these cases, spinal cord levels C2-C4 were affected (Table 3). A risk increase of myelopathy in regular users was not detected in our small sample (Odds ratio 3.2, 95% confidence interval 0.23–45.19). Only one patient presenting with right-sided hemihypesthesia and TLOC before admission exhibited left-sided brain imaging abnormalities (Three Fluid attenuated inversion revovery hyperintense, oval lesions). No evidence for autoimmune inflammation was found. Consistent with the clinical presentations, neurographic alterations were observed in all tested patients. Of the patients with available data on detailed classification of neuropathy (15 patients) 20% had axonal damage, 13.3% had demyelinating damage and 66% had mixed damage. When available (11 patients) 45.5% had pure motor neuropathy, 45.5% had sensorimotor neuropathy and one patient (4%) had pure sensory neuropathy. Somatosensory evoked potentials were abnormal in 83% of tested patients.

**Table 3:** Results of apparative diagnostics on individual patient level

Patient	MRI abnormalities	Myelopathy	Neuropathy	SSEP abnormal
1	yes (spinal)	cervical - Th10	NA	yes
2	yes (spinal)	cervical	yes	NA
3	NA	NA	yes	yes
4	NA	NA	yes	yes
5	no (spinal)		yes	NA
6	yes (spinal)	cervical	NA	yes
7	no (spinal + brain)		yes	yes
8	yes (spinal)	C2-Th8	NA	yes
9	yes (spinal)	C2	yes	yes
10	yes (spinal)	C2-C8	yes	yes
11	yes (spinal)	C2-C5	yes	yes
12	yes (spinal)	cervical, thoracal	yes	no
13	yes (spinal)	C2-C4	yes	no
14	yes (spinal)	C2-C6	yes	yes
15	yes (spinal)	yes	yes	NA
16	no		yes	NA
17	no		yes	NA
18	no		yes	NA
19	no (spinal)		yes	NA
20	yes (spinal)	C2-C7	yes	NA
21	yes (brain)		yes	NA
22	yes (spinal)	C2-C5, Th 1–2	yes	NA
23	yes (spinal)	C2-6, C7	yes	NA

Biochemical laboratory diagnostics regarding vitamin-B_12_ status were conducted on all patients (Table 1). It is noteworthy that only a minority of patients (34.8%) had abnormal vitamin-B_12_ levels. Similarly, a minor proportion of patients exhibited abnormal folate levels (21.1%). Methylmalonic acid levels were abnormal in 94.7% of patients. Furthermore, although tested in a minority of patients only, homocysteine levels were also pathological (88.9%). Preexposure vitamin-B_12_ levels were not available. Mild anemia was observed in 26.1% of cases and was asymptomatic. The mean corpuscular hemoglobin concentration was normal in all patients. One anemic female patient had a low mean corpuscular volume (78 fl.), while it was increased in one anemic patient and one patient with normal hemoglobin levels. Differential diagnoses were excluded through laboratory testing. The most common tests were infection serology (e.g. treponema pallidum, Borrelia burgdorferi, HCV, HBV, and HIV), autoimmune serology (e.g. antibodies against myelin oligodendrocyte glycoprotein, Aquaporin-4, Ganglioside, antinuclear antibodies, and anti-neutrophil cytoplasmatic antibodies), and exclusion of structural affection of the spinal cord using MRI (data not shown). Disorders of vitamin-B_12_ resorption were not routinely performed.

All patients were advised to cease inhalation of N₂O. The treatment was performed with intramuscular or intravenous injections of 1000 µg of vitamin-B_12_ for a minimum of several days, followed by less intensive oral or intramuscular substitution. Folate substitution was performed in a subset of patients. A Follow-Up was available in seven patients after four to 40 weeks (Mean 15.1, SD 15.6). Three of those had improved persisting signs and symptoms, but four did not experience a relevant improvement. No resumption of N₂O-abuse was reported.

## Discussion

The results of this retrospective study of 23 cases underscore the considerable neurological dangers associated with the recreational use of N_2_O. As previously reported for European metropolitan areas [1]; Mair, Paris, et al., [10]), there has been an alarming increase in patients presenting with neurological symptoms. The spectrum of neurological complications observed, ranging from mild sensory disturbances to severe motor deficits and cognitive changes, underscores the need for heightened awareness and prompt intervention.

Our findings corroborate those of previous studies indicating that N₂O inhalation can result in a range of neurological symptoms, predominantly due to its impact on vitamin-B_12_ metabolism. The high prevalence of sensory symptoms, including paresthesia and hypesthesia, is consistent with the known effects of SCD of the spinal cord, a condition frequently associated with vitamin-B_12_ deficiency (Mair, Paris, et al., [10] Oussalah et al., [15]. The observed motor symptoms, mainly limb weakness and hyporeflexia in over 50% of patients, further corroborate the extensive neuronal damage caused by N₂O exposure and align with previous studies [15]. Notably, autonomic symptoms were less frequently observed (13%), which may reflect the variability in individual susceptibility, differences in exposure levels, or low awareness in the assessment, as they were not routinely assessed by questionnaires or specific autonomic testing.

**Fig. 2:**
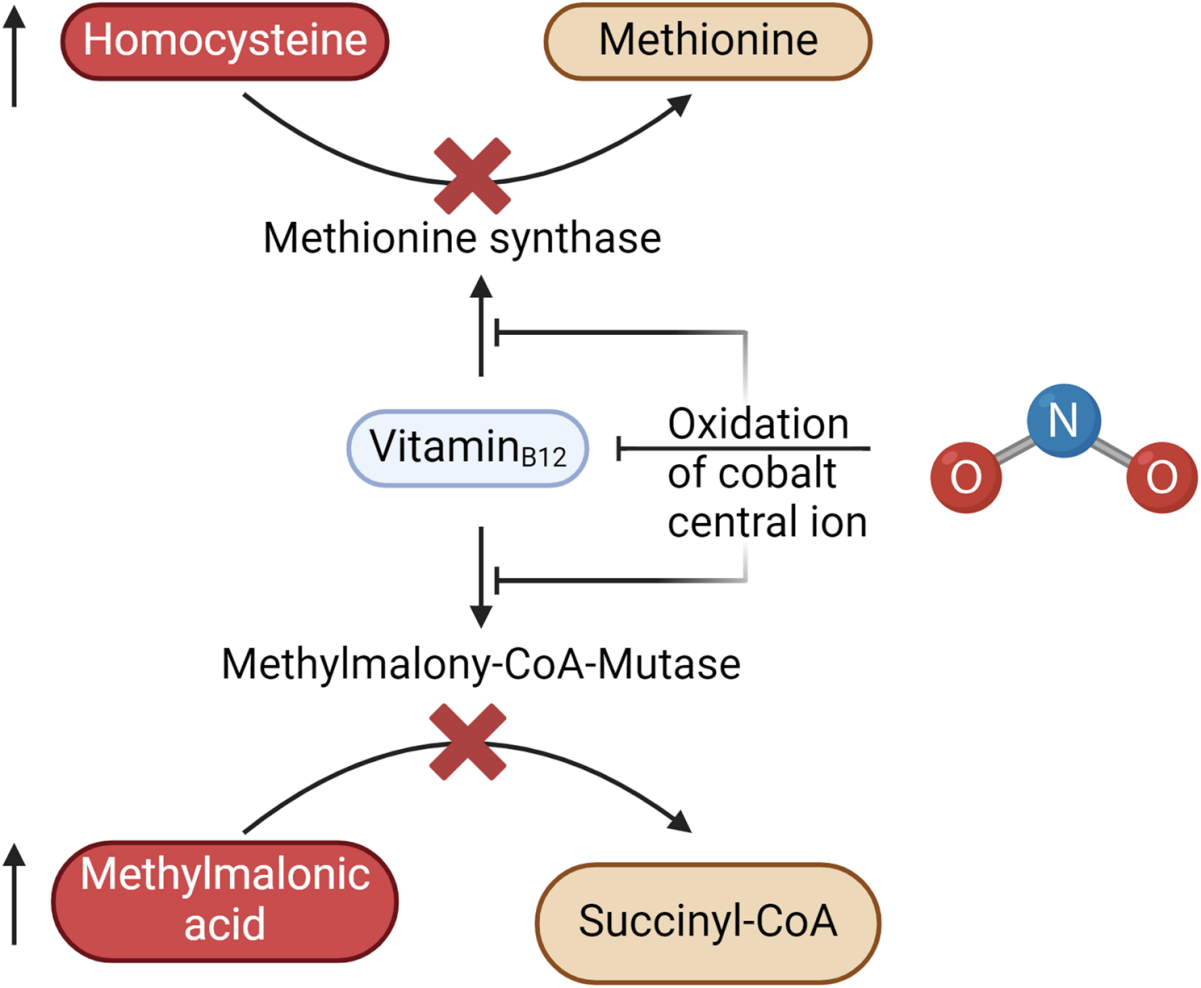
Biomarkers of N₂O-abuse. Accumulation of methylmalonic acid and homocysteine by oxidation of the cobalt central ion which leads inactivation of vitamin_B12_ that serves as a cofactor of methylmalonyl-CoA mutase and methionine synthase

Consistent with prior findings, our patients exhibited alterations in vitamin-B_12_ metabolism. Despite the clear clinical presentation of vitamin-B_12_ deficiency-related neuropathy, only a minority of patients exhibited abnormal serum vitamin-B_12_ levels (34.8%) and holotranscobalamin levels (33.3%). Holotranscobalamin, while an early marker of vitamin-B_12_ deficiency, reflects the circulating “active” B_12_ bound to transcobalamin and may not directly indicate intracellular enzymatic disruptions as quickly as methylmalonic acid and homocysteine. In contrast to vitamin-B_12_ deficiencies of other origin, which can be detected by low holotranscobalamin levels [5], this marker seems to be unaffected by N₂O-inhalation [13, 14, 16]. This discrepancy suggests that standard serum vitamin-B_12_ measurements may not be reliable indicators of functional deficiency, as previously noted (Garakani et al. 2016). In contrast, elevated homocysteine and methylmalonic acid levels appeared to be more sensitive, aligning with the understanding that functional vitamin-B_12_ deficiency can occur despite normal serum levels due to disruptions in its metabolic intracellular pathway [15]. The underlying pathomechanism is the N₂O-induced oxidation of the cobalt central ion of the biologically active methylcobalamin, which inactivates adenosylcobalamin. This leads to an inhibition of the enzymes methylmalonyl-CoA mutase and methionine synthase, whose substrates (methylmalonic acid and homocysteine, respectively) then accumulate (Fig. 2) [4]. Due to the rapid pulmonary elimination of N₂O and technically challenging detection methods such as infrared spectroscopy, direct measurement of N₂O is not established in routine care [9]. Other biomarkers of N₂O-abuse that may be changed due to the malfunction of enzymes containing vitamin-B_12_ have also been discussed, but to date have not been shown to be particularly sensitive and are not established as well [9]. Therefore, testing for the previously discussed biomarkers, methylmalonic acid and homocysteine, seems to be appropriate. It has been shown, that approximately 70% of N₂O-users tend to develop hematological abnormalities, especially low hemoglobin levels in about 50% and low mean corpuscular volumes in 40% patients [15]. Of note, in our sample such abnormalities were rarer.

Magnetic resonance imaging (MRI) revealed myelopathy in approximately one-third of patients, indicating a substantial involvement of the spinal cord in N₂O-induced neurological damage. Notably, as previously reported (Mair, Paris, et al., [10]), MRI abnormalities were most frequently observed in middle cervical spinal cord segments, suggesting a heightened susceptibility to disruptions in vitamin-B_12_ metabolism. Recent studies compared imaging features of patients with SCD related to N₂O-abuse and those with N₂O-unrelated SCD and found a more frequent cervical spinal cord involvement in N₂O users [3, 18]. Wider lesions in the N₂O-related group indicated swelling of the affected spinal cord. The authors suggested that this was due to a relatively acute effects of N₂O-abuse, whereas vitamin-B_12_ deficiency usually developes gradually [3]. In addition, N₂O has been shown to be neurotoxic by mechanisms not related to vitamin-B_12_ [17], such as *N*-methyl-D-aspartate receptor antagonism [6, 7]. The question of wether regular use of N₂O increases the likelihood of myelopathy could not be answered due to the small sample size.

The neurographic alterations and abnormal somatosensory evoked potentials (SSEPs) observed in the majority of tested patients provide further evidence of the extensive impact on the peripheral and central nervous systems. These findings are consistent with prior reports indicating that N₂O exposure can lead to demyelination and axonal damage [15].

All patients were advised to cease N₂O use and received vitamin-B_12_ supplementation, typically via intramuscular or intravenous routes. The duration of treatment varied between centers, reflecting the absence of national guidelines and variability in recovery of function. The importance of promptly addressing the underlying vitamin-B_12_ deficiency has been emphasized before, and the administration of alternate-day intramuscular injections of 1 mg hydroxocobalamin has been suggested as a standard treatment regimen [16]. While follow-up data are limited, they suggest that symptoms may improve. with appropriate treatment, indicating the potential for recovery if intervention is timely. Possible reasons for a lack of improvement may be a late begin of treatment or non-compliance.

The study’s limitations include its retrospective design and the relatively small sample size, which may limit the generalizability of the findings. Furthermore, the absence of long-term follow-up data for most patients hinders the evaluation of treatment efficacy and the potential for recovery or recurrence. The possibility of bias in case identification may also result in an underestimation of the actual number of cases, as previously discussed [16]. In addition, due to the sensorimotor focus of our analysis, neuropsychiatric symptoms, that frequently occur in N₂O-abuse [19, 20] were not assessed. Nevertheless, the study offers valuable insights into the clinical presentation and management of N₂O-induced neurological complications.

The results of the study highlight the necessity for enhanced awareness among healthcare professionals and the general public, including politicians, regarding these risks. The alarming increase in N₂O users stresses the need for public health initiatives. A British study demonstrated the feasibility of raising awareness among individuals at risk of exposure to the drug [11]. Future multicentric, prospective studies are needed to better characterize clinical, imaging and laboratory features of patients with N₂O-abuse. These should also include detailed analyses of cognitive and neuropsychiatric symptoms, as well as imaging studies of the brain and spinal cord.

A remarkable increase was observed in the number of patients presenting with neurological symptoms induced by N₂O from 2020 to 2024. This increasing trend highlights the growing prevalence of N₂O abuse and its associated neurological complications over recent years. The rising trend may be attributed to at least two factors. First, the popularity of N₂O as a recreational drug has increased, resulting in more frequent and higher dosages being inhaled by users. In turn, improved awareness among healthcare professionals regarding the neurological risks associated with N₂O may have led to enhanced recognition and reporting of such cases.

## Conclusion

The recreational use of N₂O has resulted in an increase in neurological complications, as evidenced by the rising patient numbers over the years 2020 to 2024 in five German hospitals. The interference of N₂O with Vitamin-B_12_ metabolism is the primary pathophysiological mechanism underlying these complications. The study highlights the necessity for enhanced awareness among healthcare providers and the public regarding the risks associated with the abuse of N₂O.

## Data Availability

The data supporting the findings of this study are available from the corresponding author upon reasonable request.

## Abbreviations

MRI: Magnetic resonance imaging
N₂O: Nitrous oxide
SCD: Subacute combined degeneration
SSEPs: Somatosensory evoked potentials
SD: Standard deviation
